# Linear Ballistic Accumulator Modeling of Attentional Bias Modification Revealed Disturbed Evidence Accumulation of Negative Information by Explicit Instruction

**DOI:** 10.3389/fpsyg.2019.02447

**Published:** 2019-11-07

**Authors:** Yuki Nishiguchi, Jiro Sakamoto, Yoshihiko Kunisato, Keisuke Takano

**Affiliations:** ^1^Graduate School of Arts and Sciences, The University of Tokyo, Tokyo, Japan; ^2^Faculty of Human Sciences, Sophia University, Tokyo, Japan; ^3^Artificial Intelligence Research Center, National Institute of Advanced Industrial Science and Technology, Tokyo, Japan; ^4^Department of Psychology, School of Human Sciences, Senshu University, Tokyo, Japan; ^5^Department of Psychology, Ludwig Maximilian University of Munich, Munich, Germany

**Keywords:** attentional bias modification, linear ballistic accumulator, evidence accumulation model, emotional cognition, cognitive training

## Abstract

In recent years, several attentional bias modification (ABM) studies have been conducted. Previous studies have suggested that explicit instruction (i.e., informing participants of the contingency of stimuli) enhances the effect of ABM. However, the specific working mechanism has not been identified. This is partly because reaction time (RT) data are typically reduced to an attention bias score, which is a mere difference of RT between experimental and control conditions. This data reduction causes a loss of information, as RT reflects various cognitive processes at play while making a response or decision. To overcome this issue, the present study applied linear ballistic accumulator (LBA) modeling to the outcomes (RT measures) of explicitly guided (compared to standard) ABM. This computational modeling approach allowed us to dissociate RTs into distinct components that can be relevant for attentional bias, such as efficiency of information processing or prior knowledge of the task; this provides an understanding of the mechanism of action underlying explicitly guided ABM. The analyzed data were RT-observed in the dot-probe task, which was administered before and after 3-days of ABM training. Our main focus was on the changes in LBA components that would be induced by the training. Additionally, we analyzed in-session performances over the 3 days of training. The LBA analysis revealed a significant reduction in processing efficiency (i.e., drift rate) in the congruent condition, where the target probe is presented in the same location as a negative stimulus. This explains the reduction in the overall attentional bias score, suggesting that explicit ABM suppresses processing of negative stimuli. Moreover, the results suggest that explicitly guided ABM may influence prior knowledge of the target location in the training task and make participants prepared to respond to the task. These findings highlight the usefulness of LBA-based analysis to explore the underlying cognitive mechanisms in ABM, and indeed our analyses revealed the differences between the explicit and the standard ABM that could not be identified by traditional RT analysis or attentional bias scores.

## Introduction

Selective attention to negative information or attentional bias to negative information has been repeatedly found in individuals with depression and anxiety and is also considered a key factor in the development and maintenance of these psychopathologies (Mathews et al., 1997; Mathews and MacLeod, 2005; Bar-Haim et al., 2007; Ouimet et al., 2009; Cisler and Koster, 2010; Peckham et al., 2010; Koster et al., 2011). As a measure of attentional bias, MacLeod et al. (1986) introduced the dot-probe task, which is one of the most widely used behavioral tasks to observe a bias in allocation of spatial selective attention to emotional (vs. neutral) stimuli (Kruijt et al., 2016). Some longitudinal studies have suggested that attentional bias assessed by the dot-probe task predicts a future increase in psychopathology (MacLeod and Hagan, 1992; Beevers and Carver, 2003; Ellenbogen et al., 2006; Johnson, 2009; Sanchez et al., 2013).

MacLeod et al. (2002) also developed a procedure to modify attentional bias, namely attentional bias modification (ABM), which trains participants to decrease attention to negative information. The training procedure is directly adapted from the dot-probe task with the one exception that a target probe is always presented on the opposite side to a negative stimulus. This contingency is supposed to train participants to disengage their attention from negative materials and thereby to reduce attentional bias. MacLeod et al. (2002) found that ABM suppressed negative attentional bias and reduced emotional reactivity to stress that was induced after training. Subsequent studies have replicated the finding that ABM successfully decreases stress reactivity, and also give some support for a direct intervention effect upon symptoms of anxiety (Amir et al., 2008, 2009a; Li et al., 2008; See et al., 2009; Klumpp and Amir, 2010; Eldar et al., 2012).

Another line of research suggests that the effect of ABM can be boosted when participants are explicitly instructed about the contingency between the emotional stimulus and the target in the training sessions (Krebs et al., 2010; Nishiguchi et al., 2015). In these studies, participants were explicitly instructed to attend to the opposite location when a negative word appeared on a display, while a typical (or standard) ABM instruction does not communicate the stimulus-target contingency. The results showed that the explicit instruction leads to a greater reduction in attentional bias than the standard instruction, and that this training effect transfers to performance in a different spatial attention task (Krebs et al., 2010; Grafton et al., 2014; Nishiguchi et al., 2015). Although the explicit instruction is a promising factor to accelerate the reduction of attentional bias, the mechanism underlying this promotive effect is not yet clear (see Grafton et al., 2014; Lazarov et al., 2017). Thus, the present study aimed to investigate the mechanism underlying attentional bias modulation caused by explicitly guided (compared to standard) ABM, and to specify the cognitive processes that changed through the training.

Importantly, explicitly guided ABM may reduce attentional bias more efficiently than standard ABM; however, this type of ABM (as well as the standard version) does not necessarily lead to a greater therapeutic effect. While a number of ABM studies have been published over the last 10–15 years, recent meta-analyses suggest that the effect of ABM is quite inconsistent across studies, particularly when the primary outcome is psychopathological symptoms (Hakamata et al., 2010; Hallion and Ruscio, 2011; Mogoaşe et al., 2014; Cristea et al., 2015; Mogg et al., 2017). Some studies have indeed shown a significant reduction in psychopathology after ABM training (e.g., Amir et al., 2009a, b, 2011; Wells and Beevers, 2010), whereas others failed to find such a significant therapeutic effect compared to control conditions (e.g., McNally et al., 2013; Schoorl et al., 2013). These inconsistencies in the literature resulted in a relatively small effect size as reported in recent meta-analytic studies (Cristea et al., 2015; Heeren et al., 2015; Linetzky et al., 2015). Moreover, Heeren et al. (2015) showed that explicit explanation of the therapeutic nature of the ABM can decrease the effect on psychopathology, and some studies have in fact failed to observe a significant transfer effect on psychopathology with explicitly guided ABM (Grafton et al., 2014; Nishiguchi et al., 2015). The main focus of the present study is the change in cognitive processes through ABM, but not the effects on psychopathology. As Grafton et al. (2017) discussed, aside from therapeutic effects, we can focus on changes in cognitive processes themselves to understand how and in what conditions ABM works effectively. Given that explicitly guided ABM robustly reduces attentional bias (Krebs et al., 2010; Grafton et al., 2014; Nishiguchi et al., 2015), it was more appropriate to be analyzed for the present purpose than standard ABM, regardless of the therapeutic effect. The present study focused on the analysis of the cognitive processes modified by ABM, but not on the transfer effect on psychopathological symptoms.

**FIGURE 1:**
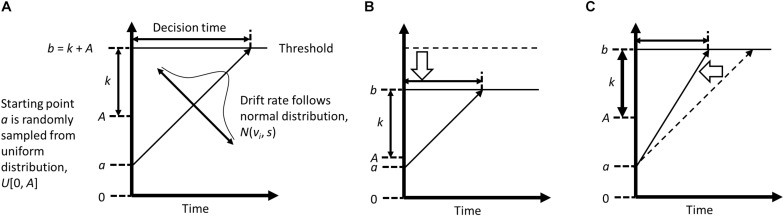
Conceptual diagrams of Liner Ballistic Accumulator model, created based on Brown and Heathcote (2008) and Annis et al. (2017). Panel **(A)** indicates a typical accumulation process that is assumed in a LBA model; Panel **(B)** (with a low threshold, caused by lower level of maximum starting evidence); and Panel **(C)** (with a high drift rate) illustrate the conditions where shorter response time is observed.

One possible strategy to infer the mechanism of action is computational modeling of reaction time (RT), which allows decomposition of RT variance into several different psychological functions that are involved in the (biased) information processing. Although attentional bias is typically indexed by mean differences in RT between conditions (i.e., congruent vs. incongruent conditions, where a target is presented in the same vs. opposite location to a preceding negative stimulus), RT contains a richer amount of information reflecting, for example, efficiency of information processing, response conservativeness, *a priori* bias, stimulus encoding, and response execution (e.g., Ratcliff, 1978). Attentional bias can be characterized by inefficient information processing in a conflicting situation (and thus RT becomes longer), which may be improved by repetitive practice to disengage attention from negative stimuli in ABM. The explicit instruction in ABM gives *a priori* bias, or prior knowledge of the contingency, which may also affect the processing efficiency and help participants to optimize their task performances. To specify the exact processes that are modified by ABM with the explicit instruction, in the current study we applied a modeling approach to RT analyses that was observed in past ABM research (Nishiguchi et al., 2015). To dissociate a single RT into different processes that are involved in that response decision, we used a linear ballistic accumulator model (LBA; Brown and Heathcote, 2005, 2008; Figure 1). In LBA, RT is considered to be the time that participants take to accumulate evidence to choose a particular response among potential response options (i.e., correct and incorrect responses in the dot-probe task). It is assumed that participants collect information from the environment after a stimulus onset, and that this information is used to make a specific response (e.g., Donkin et al., 2009). When enough evidence is accumulated to reach a threshold (or, the required amount of evidence), a decision is made, and a response emerges. Previous studies have applied the accumulation model to various cognitive processes such as lexical decision-making (Ratcliff et al., 2004; Brown and Heathcote, 2008) or recognition memory (Ratcliff, 1978; White et al., 2009). A recent study also applied an evidence accumulation model to performance in a dot-probe task (Price et al., 2019).

Although the accumulation model has several variants (e.g., the drift diffusion model; Ratcliff, 1978; Ratcliff and McKoon, 2008), most of them describe a participant’s decision-making process with four parameters: drift rate (*v*), upper limit of the starting point distribution (*A*), threshold (*b*), and non-decision time (*psi*). These parameters are often statistically estimated from RT and response accuracy (Figure 1). The drift rate represents the speed of evidence accumulation, in other words, efficiency in information processing. When attention is focused on a target stimulus, this stimulus is better processed, which typically leads to a higher drift rate. The starting point is the amount of evidence that already exists at the start of evidence accumulation, which is related to response bias. In the LBA, the starting point is assumed to vary across trials, and the upper limit of the starting point is represented by *A*, with larger values indicating greater variability in the starting point. The threshold reflects the amount of evidence that is needed to make a decision. A higher threshold indicates that participants made a response more cautiously or conservatively. Note that the absolute threshold is determined by *k* + *A*; therefore, smaller *A* indicates a lower threshold for evidence accumulation as well as less variable start points. The non-decision time is the time that was taken to encode stimuli and to execute a response.

Under the LBA framework, we hypothesized that two parameters, *v* and *A*, would be changed by the explicitly (but not implicitly) guided ABM (Krebs et al., 2010; Nishiguchi et al., 2015). First, given that the explicit instruction induces a reduction in attentional bias, *v* parameter would be affected by the training, because attention allocation to a stimulus influences information processing efficiency. The prior knowledge given by the explicit instruction may (a) disturb accumulation (low drift rate) in the congruent condition, and (b) facilitate evidence accumulation (high drift rate) in the incongruent condition, both resulting in a decreased attentional bias to negative stimuli. Second, the explicit instruction would modulate *A* parameter, which represents the variance of starting evidence and also influences the absolute threshold. This is because prior knowledge of the contingency would decrease the total amount of information that is required to make a response (i.e., a higher starting point and/or lower relative threshold; cf. Brown and Heathcote, 2008; De Loof et al., 2016). These hypotheses were tested on the dot-probe performances at the pre- and post-training assessments for the explicit vs. standard ABM. In addition, we examined participants’ performances during the training, because we predicted that awareness of the stimulus-target contingency would enhance the session-by-session progress of the training. The above described changes in LBA parameters would already be evident in the training performance, given that participants who received the explicit instruction would consciously attempt to direct their attention away from negative stimuli.

## Materials and Methods

### Dataset

The dataset (Nishiguchi et al., 2015) included responses from 40 Japanese university students without a current diagnosis of any mental illness. Among a total of 42 participants, two dropped out during the experimental sessions. All of the participants received an explanation of the experiment and completed an informed consent form before the experiment began. Half of the participants were assigned to the explicit-instruction group (*n* = 20; seven women), and the other half were assigned to the standard-instruction group (*n* = 20; eight women). Only the explicit-instruction group was informed of the stimulus-probe contingency, whilst other experimental details (e.g., content and amount of training) were identical between the two groups. All participants completed three sessions of ABM training with the modified version of the dot-probe task (one session per day). Before and after the training, participants performed the standard dot-probe task and two other behavioral tasks (i.e., the gap-overlap task and the attention network task) that were not analyzed in the present study. Participants were fully debriefed and rewarded with 3,000 Japanese yen (approximately $30 USD) at the end of the completed session.

### Materials and Tasks

#### Emotional Stimuli

A total of 160 words (80 negative, 80 neutral) from Matsumoto’s (2006) word list were selected as emotional stimuli. These stimuli were divided into two sets, each including 40 negative and 40 neutral words. A one-word set was used for pre-training assessment and ABM training; the other set was used for post-training assessment only.

#### Dot-Probe Task

The dot-probe task was used in the pre-training and post-training assessment. Each trial began with a fixation cross presented at the center of the screen for 500 ms. Two words appeared at the left and right of the fixation cross for 1500 ms. Upon the removal of the words, a target probe (white square) appeared at the left or right of the fixation cross. Participants had to indicate the location of the target by a key press within 1000 ms of the onset of the target. If a response was not made within this time limit, it was regarded as an error response, and the next trial was automatically started.

There were three types of trial according to the cue-target contingency. In the congruent trials, a negative-neutral word pair appeared on a screen, following which a target probe was presented at the place that the negative stimulus occupied. In the incongruent trials, the target was presented at the opposite location to the negative stimulus. In the neutral trials, the stimulus pair was both neutral words. In one test session, 80 neutral, 40 congruent, and 40 incongruent trials were presented in random order, and these trials were separated into two blocks of 80 trials.

The attentional bias index was calculated with the following formula: [(average RTs on incongruent trials) − (average RTs on congruent trials)]/(average RTs on all trials). Higher scores indicate stronger attentional bias to negative stimuli.

### Attention Bias Modification (ABM) Procedure

The modified version of the dot-probe task which was used for the ABM training followed the exact same procedure as the standard dot-probe task that was used in the assessments. The only exception was that there were no congruent trials in the modified version. Each training session consisted of 80 neutral trials and 80 incongruent trials. We expected that if the training consisted only of incongruent trials, participants might learn to just search for a neutral word (without attending to a negative word) to respond to the target efficiently. Because this does not improve attentional disengagement from negative stimuli, we implemented neutral trials to prevent participants from learning this “attend-neutral” strategy. These trials were divided into two blocks and presented in a random order. Participants completed three training sessions (one session per day), resulting in a total of 240 neutral trials and 240 incongruent trials.

Participants received either the explicit or standard instruction. Participants in the explicit-instruction group were told that the target always appeared on the opposite side to a negative word. Thus, participants were expected to attend to the side opposite a negative word when the participants find one. On the other hand, participants assigned to the standard-instruction group were not informed of the cue-target contingency and were only told that the target appeared on either the left or right side of the central fixation after a pair of words were presented.

### Linear Ballistic Accumulator Model

In an LBA model (Brown and Heathcote, 2008), the evidence accumulation process is represented by four free parameters (*v*, *A*, *b*, *psi*) as described in Figure 1. The drift rate (*v*) codes efficiency of evidence accumulation, which starts at point *a*, sampled at each trial from a uniform distribution, *U*[0, *A*]. The threshold *b* denotes the amount of evidence that is needed to make a response. We followed the formulation of Annis et al. (2017), where *b* is determined by the relative threshold (*k*) and the maximum starting point (*A*). With these parameters, the time to make a response (the decision time) is defined as the distance between the starting point and threshold divided by the drift rate. Observed RT normally comprises the decision time and the time that is spent for perceptual and motor processing, which are referred as non-decision time, *psi*. It may be also noteworthy that LBA can be applied regardless of the number of response alternatives. An LBA model assumes that each response option has one accumulator; thus, when there are *N* (*N* = 1, 2, 3, …) alternatives for a possible response, *N* accumulators are assumed. Unlike other accumulation models such as the drift-diffusion model (Ratcliff, 1978), LBA assumes that evidence is accumulated independently across response options. This means that there is no limit for the number of accumulators that can be included in a model (Brown and Heathcote, 2008). For example, Zhang et al. (2012) assumed three accumulators (corresponding to three response alternatives for one trial) in the LBA model. This feature also allows us to assume only one accumulator in a model, which codes the accumulation process solely for correct responses. The accumulator for incorrect responses was not assumed in our analyses, because the response accuracy was quite high in the current data (>99% in pre-tests, post-tests, and training sessions) and it was not possible to estimate LBA parameters for error responses.

The LBA model was applied to the entire group of participants, with the ABM-instruction differences as a between-participant factor, and the dot-probe trial types and assessment time as within-participant factors. The parameters could vary across the trial types and the explicit- and implicit-instruction groups. To estimate these parameters, we used the Hamiltonian Monte Carlo (HMC) algorithm in the present study. The HMC is a parameter estimation method based on Bayesian statistics, and is a kind of Markov Chain Monte Carlo (MCMC) algorithm. The MCMC is a sampling algorithm to generate sample distributions according to the prior distribution of the parameters. Parameters are continuously shifted in accordance with some rule until the sample distribution becomes enough close to the target distribution. The HMC algorithm applies the basic principle of Hamiltonian mechanics as the rule. Compared to traditional MCMC techniques, the HMC improves the efficiency of parameter estimation, and requires shorter chains (Hajian, 2007). In the present study, the HMC algorithm (iteration = 4000, warmup = 2000, thinning = 4) was conducted to obtain posterior distributions of pre-to-post changes in each LBA parameter for the 2 (instruction) × 3 (dot-probe trial type) conditions (Appendix). Four HMC chains were run to evaluate the convergence, which meets the Gelman-Rubin’s criteria (Gelman and Rubin, 1992; $\hat{R}$ close to 1). These analyses were performed using R (version 3.4.0, R Core Team, 2017) and the rstan package for HMC (Carpenter et al., 2017; R and Stan code used in the present study are uploaded here: https://osf.io/u5cq6/files/).

## Results

### Performances in the Pre- and Post-test Assessments

#### Descriptive Statistics

Average RTs for each condition in the dot-probe task, excluding trials with errors or extremely short or long RTs^1^ (average RTs ± 2*SD*), are shown in Table 1. As reported in Nishiguchi et al. (2015), the results of ANOVA (time × group) revealed that the attentional bias index in the explicit instruction group was significantly lower for the post-test session, *d* = 1.07, while there was no significant difference in the standard instruction group, *d* = 0.15 (*F*(1, 38) = 8.47, *p* < 0.01, for the interaction between time and group).

**TABLE 1:** Mean RTs (ms) and standard errors (*SE*) for each condition in the dot-probe task for the pre- and post-test assessments.

	**Explicit (*n* = 20)**				**Standard (*n* = 20)**			
		
	**Pre**		**Post**		**Pre**		**Post**	
				
	***M***	***SE***	***M***	***SE***	***M***	***SE***	***M***	***SE***
Incongruent	382	10	355	11	380	8	359	7
Congruent	380	10	365	12	379	9	356	7
Neutral	380	10	362	12	378	9	358	7

#### Analyses With LBA Modeling

**FIGURE 2:**
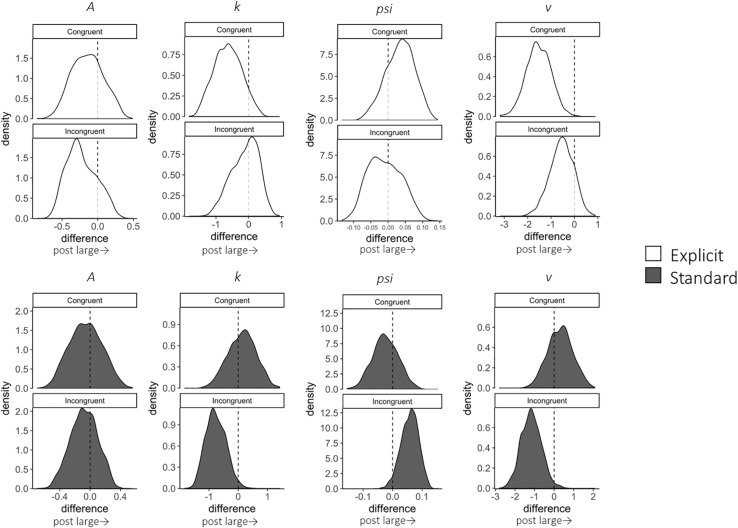
Change in LBA parameter distributions in congruent and incongruent trials from pre- to post-test, calculated by subtracting pre-test distributions from post-test distributions. Among the four parameters (*A*, *k*, *psi*, *v*), *A* parameter represents the maximum amount of initial evidence, and *k* parameter represents relative threshold (when *b* parameter represents the threshold, *b* – *A*). *psi* parameter represents the non-decision time, and *v* parameter represents the drift rate. White distributions are those of the explicit group and gray ones are of the standard group.

A total of 12,800 trials of the pre- and post-training dot-probe task, across all participants and trial types, were submitted to LBA modeling.^2^ Satisfactory convergence was found for all estimated parameters according to Gelman-Rubin statistics: all $\hat{R}$ < 1.1; all effective sample size (ESS)/total samples > 10%; all MCSE/*SD* < 10%. We excluded 124 trials with errors or extremely short response times (<200 ms) to improve the accuracy of estimation with LBA. Pre-to-post changes in LBA parameters (subtracting post- from pre-estimates) were calculated for each combination of the conditions (trial type: congruent, incongruent, and neutral; instruction: explicit, standard). Density plots are shown in Figure 2 for each group and trial type. In addition, we calculated group differences in changes in LBA parameters (subtracting pre-post changes in the standard group from those in the explicit group) to highlight the specific changes for the explicit but not standard instruction (Table 2).

**TABLE 2:** Changes in LBA parameters between pre- and post-assessments.

		**Explicit instruction (Post-Pre)**		**Standard instruction (Post-Pre)**		**Group difference**	
				
		**EAP**	**95% CI**	**EAP**	**95%CI**	**EAP**	**95%CI**
Congruent	*A*	–0.14	[−0.55,0.29]	–0.06	[−0.45,0.35]	–0.09	[−0.66,0.53]
	*k*	–0.64	[−1.41,0.17]	0.13	[−0.79, 1.00]	–0.77	[−1.93,0.45]
	*psi*	0.04	[−0.05, 0.11]	–0.02	[−0.11, 0.06]	0.06	[−0.06, 0.17]
	*v*	–1.53	[−2.55, −0.53]	0.31	[−0.91, 1.54]	–1.84	[−3.40, −0.22]
Incongruent	*A*	–0.22	[−0.57, 0.20]	–0.08	[−0.43, 0.26]	–0.15	[−0.66,0.40]
	*k*	–0.12	[−0.98, 0.56]	–0.76	[−1.38, −0.06]	0.65	[−0.44,1.64]
	*psi*	–0.01	[−0.09, 0.08]	0.06	[0.00, 0.11]	–0.07	[−0.17, 0.04]
	*v*	–0.54	[−1.55, 0.37]	–1.17	[−2.16, −0.13]	0.63	[−0.85, 2.02]
Neutral	*A*	–0.08	[−0.39, 0.21]	–0.03	[−0.34, 0.29]	–0.05	[−0.51, 0.37]
	*k*	–0.78	[−1.27, −0.24]	–0.71	[−1.26, −0.08]	–0.08	[−0.89, 0.70]
	*psi*	0.06	[0.01, 0.10]	0.06	[0.00, 0.11]	0.00	[−0.08, 0.07]
	*v*	–1.46	[−2.09, −0.76]	–0.93	[−1.70, −0.10]	–0.53	[−1.59, 0.51]

*Group differences in LBA parameters were calculated by subtracting the pre-post changes in the standard group from those in the explicit group. LBA, Linear Ballistic Accumulation model; EAP, expected-*a posteriori*, which indicates the parameter for each estimated posterior distribution; *v*, drift rate; *A*, upper limit of the starting point distribution; *k*, relative threshold*; psi*, non-decision time.*

In terms of the training effect, significant group differences in the extent of pre-to-post changes were found for the *v* (the drift rate) parameter; expected-*a posteriori* (EAP) estimate (EAP_post–pre_) = −1.840, 95% Credible Interval (CI) [−3.395, −0.217], which did not include zero. The explicit group showed a reduction in the *v* parameter for congruent trials; EAP_post–pre_ = −1.528, 95% CI [−2.545, −0.528], which did not include zero in the interval. This result indicates that the speed of evidence accumulation was decreased when the target was presented at the same location as the negative stimulus. On the other hand, this reduction in the *v* parameter was not found in the standard group, EAP_post–pre_ = 0.312, 95% CI [−0.907, 1.539].

Although there were no other significant group differences, some within-group changes were found. Firstly, reduced *v* parameters were also present in incongruent trials in the standard group; EAP_post–pre_ = −1.168, 95% CI [−2.160, −0.131], which did not include zero. However, there was no change for the explicit group; EAP_post–pre_ = −0.543, 95% CI [−1.550, 0.371]. Moreover, the standard group showed a significant reduction in the EAP of the *k* (the threshold) parameter in the incongruent condition; EAP_post–pre_ = −0.762, 95% CI [−1.376, −0.060], which did not include zero. On the other hand, the explicit group did not show a significant *k*-change; EAP_post–pre_ = −0.116, 95% CI [−0.979, 0.563].

### Performance Changes Across the Training Sessions

#### Descriptive Statistics

Average RTs (and *SE*) for each condition in each training session are shown in Table 3. There were a total of 19,200 trials over the 3 days of ABM training. We excluded 1,406 trials with errors and extremely short response times (<200 ms) to improve the accuracy of estimates with LBA.

**TABLE 3:** Mean Reaction Times for each condition in training task.

	**Explicit instruction (*n* = 20)**						**Standard instruction (*n* = 20)**					
		
	**Day 1**		**Day 2**		**Day 3**		**Day 1**		**Day 2**		**Day 3**	
						
	***M***	***SE***	***M***	***SE***	***M***	***SE***	***M***	***SE***	***M***	***SE***	***M***	***SE***
Incongruent	292	8	277	7	277	7	350	7	348	6	340	7
Neutral	356	8	347	7	336	7	350	7	348	6	344	5

#### Analyses With LBA Modeling

**FIGURE 3:**
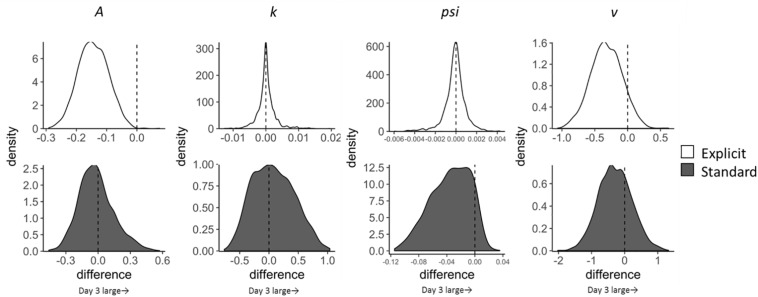
Change in LBA parameter distributions for incongruent trials from Day 1 to Day 3 calculated by subtracting Day 1 parameter distribution from Day 3 distribution. Upper four distributions are those of the explicit group and lower ones are of the standard group.

Temporal changes in LBA parameters across the training sessions (Day 1–3) were calculated for each condition (incongruent and neutral trials; explicit and standard instruction) by subtracting parameters on day 1 from those on day 3 (Figure 3). Satisfactory convergence was found for all estimated parameters according to Gelman-Rubin diagnosis: all $\hat{R}$s < 1.1; effective ESS/total samples > 10%; MCSE/*SD* < 10%.

As for the pre-post analyses, we computed group differences in changes in LBA parameters (subtracting Day 1–3 change scores in the standard group from those in the explicit group). The results, however, showed that none of the parameters had significant group differences in the extent of changes. Although group differences were not clear, we found that the *A* parameter decreased in incongruent trials in the explicit group; EAP_Day 3__–__1_ = −0.144, 95% CI [−0.243, −0.045], which did not include zero. This reduction in *A* can be interpreted as a reduction of variance in the starting point; in other words, participants in the explicit group acquired a stable state of preparation for the target that appeared at the opposite location to the negative stimuli throughout the training session. Moreover, since the threshold is represented by *k* + *A* in the present LBA model, reduction in the *A* parameter also indicates a reduction in the threshold, which leads to reduced RT, congruent with the results shown in Table 3. The standard group did not show this reduction in the *A* parameter; EAP_Day 3__–__1_ = 0.015, 95% CI [−0.355, 0.296].

**FIGURE 4:**
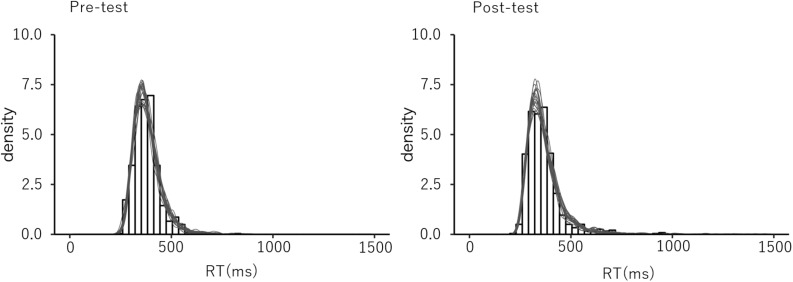
The example of the post predictive check. The observed data distribution and the generated distribution in the post predictive check in the congruent trials in the pre- and the post-test sessions for the explicit group.

To test the goodness of fit of the estimated model, we conducted a posterior predictive check (Gelman et al., 1996; Gelman and Shalizi, 2013). Based on the estimated LBA parameters, RT distributions were generated and compared to the observed RT data for replicability. The results showed that the distribution of the generated RTs were sufficiently similar to that of the observed data for all conditions, which suggests a good fit of the estimated model (Figure 4).

## Discussion

In this study, we examined the training effect of explicitly and standardly guided ABM by using the LBA model. The computational approach enabled us to clarify the cognitive processes that are modulated by explicit ABM compared with standard ABM, which could not be identified by mere RT data or traditional attention bias scores. Performances in the standard and modified (i.e., for training) versions of the dot-probe task were collapsed into LBA parameters including the starting point (*A*, maximum amount of initial evidence and absolute threshold) and the drift rate (*v*, speed of evidence accumulation). The results of LBA modeling revealed that the drift rate decreases in the congruent but not in the incongruent trials after completing the explicitly, but not standardly, guided ABM. Moreover, although there were no significant group differences, the *v* and *k* parameters for incongruent trials decreased in the standard group. Through the training session, the *A* parameter decreased in the explicit group but not in the standard group.

The significant reduction in the drift rate suggests that the explicit instruction causes a slowdown in evidence accumulation for the congruent trials. This result is consistent with our first hypothesis that explicitly guided ABM influences the accumulation speed and efficiency of information processing. When being explicitly instructed, participants consciously practice directing their attention away from a negative stimulus. This awareness of the incongruent contingency would be reinforced via repeated practice in the training session (which have no congruent trials), and could also facilitate response habituation to the incongruent trials. Such beliefs and training may disturb the accumulation of evidence in the congruent condition at post-training assessment, as participants would direct their attention away from negative stimuli even in the congruent trials. This reduction in the drift rate can be explained by Inhibition of Return (IOR; Posner, 1980; Klein, 2000; Hilchey et al., 2014), which may have occurred if participants attended to negative stimuli once and then instantly disengaged their attention from it. IOR causes difficulty in re-orientating attention to a location where attention was allocated shortly before it was disengaged. After the intensive and conscious training of attentional disengagement, IOR could prevent participants from re-orienting attention to the location where a negative stimulus appeared.

Interestingly, the drift rate in the incongruent trials was not increased by the explicitly guided ABM, which is not consistent with our hypothesis. Given that the explicit instruction informed participants of the contingency between the target and negative stimulus, this prior knowledge could have enhanced evidence accumulation in the incongruent condition, but may have rather impeded accumulation in the congruent condition. Although we do not have a clear explanation for this null result, it may be explained by the overlearning of attentional disengagement through the ABM. No attentional bias was found in the pre-training assessment, which means that the RTs in congruent and incongruent conditions were not significantly different. Thus, it can be inferred that attentional disengagement from negative stimuli was not difficult for the present sample (non-clinical students) and there was no room to induce a significant change in their responses to incongruent trials. Instead, participants may have (over) learned attentional disengagement from negative stimuli, which automatically guides their attention away from the negative stimulus even in congruent trials (so that evidence accumulation is delayed).

On the other hand, the standard group did not show a significant change in the drift rate in the congruent condition. This implies that target processing in the congruent trials is neither enhanced nor inhibited by standardly guided training. The standard ABM does not instruct participants to attend or not to attend to negative stimuli, which may have only partially promoted attentional disengagement from negative stimuli. Therefore, IOR may not have taken place in the congruent trials at the post-training assessment. Additionally, though there was no change in congruent trials, the *v* and *k* parameters were decreased in incongruent trials in the standard group. The decrease in *k* means that the required amount of information to make a response was decreased over the training period, which typically leads to faster overall RTs if other parameters are unchanged. On the other hand, the reduction in *v* suggests delayed evidence accumulation, i.e., slower overall responses, after the training. These results seem to suggest that the standard ABM may change the way that information is processed, even though the changes are not visible in mere RTs or bias indices. However, because there was no group difference in the *v* and *k* parameters, these results should be interpreted carefully, and further investigations and replications are warranted.

On top of the pre- and post-training assessment, we focused on performance changes over the training sessions. The results indicated that *A*, which represents the starting point variability and the absolute threshold, decreased over the training sessions in the explicit (but not standard) group. With a smaller *A* value, the starting point (i.e., *a*) distributes in a narrower range (i.e., having smaller variance), and the absolute threshold also becomes lower. The evidence accumulation starts almost always from the same point, and participants make a response with a smaller amount of evidence. Participants in the explicit group were repeatedly trained with prior knowledge about the cue-target contingency. This consequently created a stable preparatory state for the incongruent target in the training task, which might have resulted in a decreased *A* parameter (or decreased variance in the starting point). Not much evidence was needed for the participants to make a response in the last session of training, because they had learned the task to saturation. The relative threshold, *k*, may also be changed by prior knowledge or the explicitly guided ABM (since the absolute threshold is determined by *k* + *A* in LBA). However, when response speed is emphasized in a task (participants must make a response as quickly as possible), the threshold is fixed to, or near, the upper limit of the starting point distribution (i.e., the *A* parameter; Brown and Heathcote, 2008). Since participants were instructed to make a response as fast as possible in the present tasks, the variance of the threshold might have been completely absorbed by *A* but not *k*, and so the effect of prior knowledge was more prominent on the *A* parameter.

The present study revealed the parameters that were changed by explicitly guided ABM, which is a possible cause of efficient attentional bias reduction. However, we should carefully interpret the clinical implications because although previous studies have suggested that explicitly guided ABM efficiently improves attentional bias, the effect on psychopathological symptoms and stress reactivity are less evident (e.g., Grafton et al., 2014). These contradictory findings may question the hypothesized mechanism; i.e., that ABM exerts the therapeutic effect on symptomatology specifically via reduced attentional bias. Future research must clarify the boundary conditions that predict whether ABM shows a clinically meaningful effect. One of the boundary conditions may be an overall reduction in attentional bias (as theories predict), which may be combined with changes in other specific cognitive processes. Given that our explicitly guided ABM was successful in reducing attentional bias, but not depressive symptoms (Nishiguchi et al., 2015), a change in drift rate may not be the sole condition necessary to ensure positive effects on symptomatology. We believe that LBA is a useful tool to understand the mechanism of attentional-bias reduction through ABM, especially if our findings are compared with the results of other ABM trials that successfully reduced symptomatology in terms of parameter changes in LBA.

The present study has several procedural limitations, many of which were already discussed elsewhere (Nishiguchi et al., 2015), such as the short-term and minimal extent of training, there being no follow-up assessment, and a small sample size. Here, we focus on LBA-specific issues and limitations that should be noted when interpreting our results. The four parameters estimated in LBA (i.e., *A*, *k*, *psi*, and *v*) often mutually correlate with each other, which sometimes makes it difficult to reliably distinguish between each process. One possible solution for this issue is to have a large enough sample size (Visser and Poessé, 2017). In our analysis, therefore, we applied the LBA model to the trials across all participants, allowing the parameters to vary across conditions and time, but not across participants; this approach led to a crucial limitation in that the model cannot estimate the parameters for each individual participant. If a larger number of observations were available, the individual differences in each LBA parameter change could be estimated. This provides another interesting opportunity for further investigation, for example, whether the correlations between the parameters change over the training sessions and the performance changes from the pre- to post-training assessment. Another issue is that we did not include error trials in the analyses. As there were numerous error trials in the present dot-probe data (accuracy = 99%), it was not possible to estimate the accumulator for incorrect responses. Although the number of alternatives is not restricted in LBA, it is hoped that this limitation could be resolved by applying the model to data including more errors, such as data from clinical samples. A further issue in the present study is that the posterior distributions of the parameters appear to be non-normally distributed. Although we do not have a clear explanation or interpretation for this, our choice of priors (i.e., weakly informative distributions) may have influenced this skewness. There was no previous example of LBA modeling of ABM which we could refer to in the setting of the prior distribution, thus we assumed a weakly informative distribution (following the recommendation of the Stan Development Team, 2019). If data on the distribution of LBA parameters in ABM were available in the future, other options for prior distribution may be selected, which could lead to normality in the posterior distribution of the parameters.

In conclusion, the present study is, to the best of our knowledge, the first that has applied LBA to analyze and model the effect of ABM. The LBA approach showed that explicitly guided ABM slows down evidence accumulation in congruent trials of the dot-probe task, possibly because the training promotes attentional disengagement from negative stimuli. The explicit instruction gave participants prior information about the cue-target contingency, which could enhance the learning of attentional disengagement from negative stimuli through the training sessions. Although the non-clinical nature of the sample limits the clinical implications of our findings, the computational modeling approach used could deepen our understanding of processes that can be modulated by ABM. Finally, although the present study is the first attempt to apply LBA to the analysis of ABM, computational approaches to attentional bias are getting attention (e.g., Pe et al., 2013; Price et al., 2019). Future research needs to apply LBA and other accumulator models to clinical trials of ABM, which would reveal what process changes take place behind the bias and symptom reductions in ABM.

## Data Availability Statement

The datasets generated for this study are available on request to the corresponding author.

## Ethics Statement

Ethical review and approval was not required for the study on human participants in accordance with the local legislation and institutional requirements. The patients/participants provided their written informed consent to participate in this study.

## Author Contributions

YN and KT conceived the study and conducted the original experiment. JS and YK planned and conducted the analysis. YN, JS, YK, and KT interpreted the results, wrote the manuscript, and approved the final version of the manuscript.

## Conflict of Interest

The authors declare that the research was conducted in the absence of any commercial or financial relationships that could be construed as a potential conflict of interest.

## Footnotes

1 To keep the consistency with the previous study (Nishiguchi et al., 2015), we used mean ± 2*SD* as the exclusion criteria for this bias-score analysis.

2 We initially applied a LBA model to each individual participant, however, the Markov chain Monte Carlo (MCMC) chains did not converge to a stationary distribution. Therefore the model was designed to estimate parameters for each group and condition, but not for each participant.

## Appendix

As we were not aware of previous research on appropriate priors for Bayesian estimation of LBA models, we assumed that each parameter follows a weakly informative prior (i.e., half-Normal distribution), for the *i*-th accumulator and *j*-th trial, as follows:

$$A_{\text{ij}}∼half\text{-}Normal\left(0,10\right),$$

$$k_{\text{ij}}∼N\left(0,10\right)T\left(0\right),$$

$$v_{\text{ij}}∼N\left(0,10\right),$$

$${psi}_{\text{ij}}∼N\left(0,10\right)T\left(0\right).$$
